# Radiation-Induced Helium Bubbles in Metals

**DOI:** 10.3390/ma12071036

**Published:** 2019-03-28

**Authors:** Shi-Hao Li, Jing-Ting Li, Wei-Zhong Han

**Affiliations:** Center for Advancing Materials Performance from the Nanoscale (CAMP-Nano), State Key Laboratory for Mechanical Behavior of Materials, Xi’an Jiaotong University, Xi’an 710049, China; lsh4007025@gmail.com (S.-H.L.); lijingting235@sina.com (J.-T.L.)

**Keywords:** helium bubbles, bubble evolution, interfaces, radiation hardening, helium embrittlement

## Abstract

Helium (He) bubbles are typical radiation defects in structural materials in nuclear reactors after high dose energetic particle irradiation. In the past decades, extensive studies have been conducted to explore the dynamic evolution of He bubbles under various conditions and to investigate He-induced hardening and embrittlement. In this review, we summarize the current understanding of the behavior of He bubbles in metals; overview the mechanisms of He bubble nucleation, growth, and coarsening; introduce the latest methods of He control by using interfaces in nanocrystalline metals and metallic multilayers; analyze the effects of He bubbles on strength and ductility of metals; and point out some remaining questions related to He bubbles that are crucial for design of advanced radiation-tolerant materials.

## 1. Introduction

**Figure 1:**
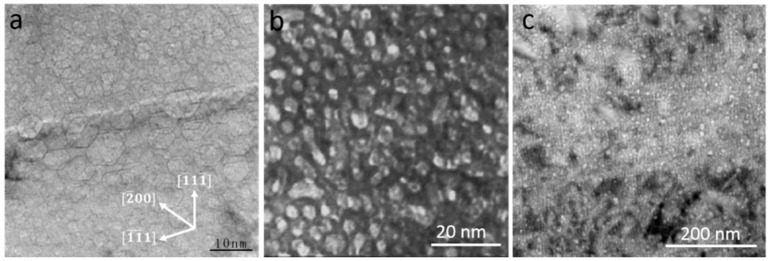
Dense He bubbles after He irradiation in: (**a**) Al [10]; (**b**) tungsten [11]; and (**c**) Zr [12]. Reprinted with permission from [10]; Copyright 2015 Elsevier; Reprinted with permission from [11]; Copyright 2000 Elsevier; Reprinted with permission from [12]; Copyright 2017 Elsevier.

Nuclear energy is playing an increasingly important role because fossil fuels are gradually running out. Nuclear energy currently provides about 13% of electrical power worldwide [1]. However, materials deployed in fission, spallation and fusion systems suffer from intense high-energy neutron radiation [1]. Continuous collision cascades produce massive vacancy clusters and interstitials in nuclear component materials, which promotes formation of various defects, such as dislocation loops [2,3], voids [4,5], stacking fault tetrahedral (SFT) [6,7] and so on. In addition, (*n*, α) reactions produce abundant helium (He) atoms in materials. As He has extremely low solubility in metals, it tends to accumulate and precipitate into nanoscale He bubbles in nuclear structure materials [8,9,10,11,12,13,14]. Figure 1 shows typical examples of transmission electron microscope (TEM) images of He bubbles formed in Al [10], tungsten [11] and Zr [12,13,14] after He^+^ ion irradiation. 

A comprehensive understanding on the dynamic evolution of He bubbles and their effects on mechanical properties of nuclear structure materials over the lifetime remains as one of the key issues in nuclear industry. He bubbles are found to drastically deteriorate mechanical properties of metals, manifested as swelling [15,16], blistering [17] and embrittlement at high temperature [18,19]. Among these degraded properties, He-induced embrittlement has attracted particular attention since it causes catastrophic fracture in metals, particularly at high temperature [18,19]. It has been well demonstrated that even extremely low overall He concentration can lead to He embrittlement via formation of He bubbles along grain boundaries (GBs) [18,19]. In view of this, several decades of investigations have been conducted to unveil He behaviors and underlying mechanisms for He-induced degradation in metals [8,9,10,11,12,13,14,15,16,17,18,19,20,21,22,23,24]. Both experiments and atomic simulations are adopted to investigate He bubble formation, dynamic evolution and their effects on mechanical properties of metals. 

In this review, we briefly summarize previous studies on He bubbles in metals. Section 2 reviews research on He bubble nucleation, growth and coarsening. Section 3 summarizes He bubble behaviors in nanocrystalline metals and metallic multilayers and emphasizes the important role of interfaces. Section 4 overviews the He-induced hardening in single-phase metals and metallic multilayers. Finally, we summarize the main results and discuss the critical questions remained.

## 2. Helium Bubble Nucleation, Growth and Coarsening

As mentioned above, due to their extremely low solubility, He atoms tend to agglomerate into He bubbles in metals. A comprehensive understanding on bubble nucleation, growth and coarsening is crucial to evaluate the role of bubbles in metals. The formation of He bubbles can be divided into homogeneous or heterogeneous nucleation. In this section, we focus on the mechanism of homogeneous bubble nucleation. In general, bubble nucleation, growth and coarsening are controlled by the concurrent operation (including diffusion, clustering, dissociation and recombination, etc.) of He atoms, vacancies and interstitials. However, limited spatial resolution and temporary resolution of various instruments hinder the direct real-time atomic-scale observation of bubble formation, which prevents unveiling the mechanisms underlying bubble nucleation, growth and coarsening. Studies on this issue using computer simulation methods [25,26,27,28,29,30,31,32,33,34,35,36,37,38] have been conducted in past decades. Because steels are widely used as nuclear reactor components and tungsten is regarded as the main candidate material for plasma facing materials (PFM) in future fusion reactors, profuse studies are focused on behaviors of He atoms, vacancies and interstitials in Fe and tungsten [25,26,27,28,29,30,31,32,33,34,35,36,37,38]. Generally, body-centered-cubic (BCC) metals have attracted more attention due to their better radiation resistance than face-centered-cubic (FCC) metals.

### 2.1. He–V Clusters

**Figure 2:**
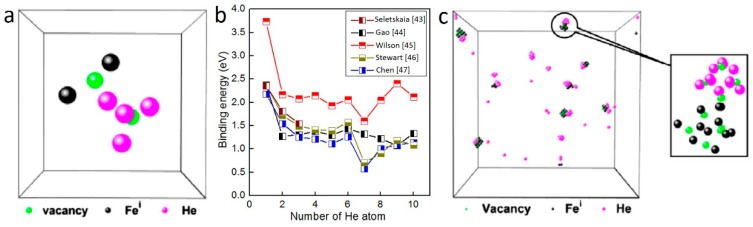
(**a**) Schematic illustration of He–V cluster in Fe [27]; (**b**) the dependence of binding energy of interstitial He to He–V cluster on the number of He atom in He–V cluster [43,44,45,46,47]; and (**c**) He clusters and other defect distribution in Fe containing 125 He atoms. The inserted image is the detailed arrangements of a He-interstitial cluster [27]. Reprinted with permission from [27]; Copyright 2013 Elsevier.

Rimmer and Cottrell [39] investigated He behaviors in metals and demonstrated that He accumulation can be ascribed to two reasons: first, the interstitial He has low migration energy in metals; and, second, the substitutional He is easily trapped at vacancies because of their high binding energy. Several studies [29,34,37,40,41,42] point out that interstitial He is energetically favorable to occupy the tetrahedral interstitial site (TIS) with low formation energy in BCC metals. Becquart [41] evaluated the formation energies of interstitial He in tungsten using ab initial calculations and found that the formation energies of interstitial He at TIS is 6.18 eV, which is slightly lower than that in octahedral interstitial sites (6.40 eV). Other studies [42,43,44,45] propose a similar trend that TISs are preferable He traps. Notably, due to the low migration energy [27,31], interstitial He in tetrahedral sites and small He clusters (mainly He_2_) tend to diffuse easily in metals. These isolated He and small He clusters are easily trapped by vacancies and form a sphere-like configuration of He–V clusters with low mobility [25,26,27,28,29,30,31,32,33,34,35,36,37,38,39,40,41,42,43,44]. Consequently, He–V clusters deliver high binding energy, performing as sinks for interstitial He and small He clusters and giving rise to He bubble nucleation in metals [43,44,45,46,47]. Figure 2a schematically illustrates an isolated He–V cluster containing four He atoms in Fe, which can be regarded as embryo of He bubble [27]. Fu et al. [48] showed that small He–V clusters (up to four helium atoms) will lead to bubble nucleation in initial vacancy-free lattices. The binding energy for additional He atoms to combine with He–V cluster in Fe is plotted in Figure 2b [43,44,45,46,47]. The binding energy of an interstitial He to He–V clusters is initially high, then it decreases sharply with increasing He atoms, and keeps almost constant with a value of about 1.3 eV with further increasing He atoms. It should be noted that the binding energy reaches a local peak value with six He atoms in He–V cluster, and an additional He induces significant reduction in binding energy. He–V clusters containing six He deliver compact octahedral shape with He located at corners and the vacancy at the center [44]. An extra He will trigger the kick-out of Fe around the cluster, leading to reduced binding energy [44]. The kick-out mechanism is discussed below. Finally, as shown in Figure 2c, the matrix in molecular dynamics (MD) simulations is quenched to 0 K, followed by annealing at 800 K for 1.2 ns, and then a few He–V clusters are formed in Fe containing 685 appm He [27]. 

### 2.2. Kick-Out Mechanism

**Figure 3:**
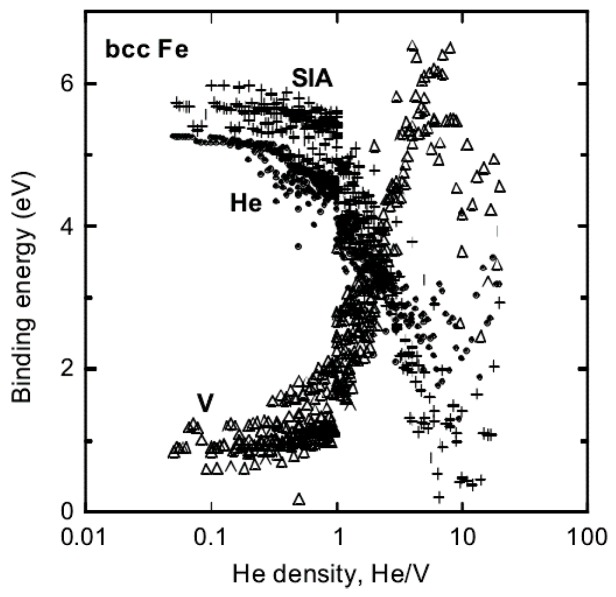
The variation of binding energies of a vacancy, an interstitial He atom and a SIA to He–V cluster with different He density in Fe. Reprinted with permission from [25]; Copyright 2003 Elsevier.

With gradual accumulation of He in He–V clusters, the pressure induced by He–V clusters on surrounding lattice will increase significantly, and then the matrix atoms surrounding He–V clusters will be pushed out due to the high pressure, producing self-interstitial atoms (SIA) and vacancies (SIA-V pairs). That is how He–V clusters lead to kick-out of matrix atom and formation of Frenkel pairs. By investigating the dependence of the binding energy of He atoms, vacancies and interstitials on He density (defined as He/V ratio) in He–V clusters using MD simulations, Morishita et al. [25] described the kick-out mechanism quantitatively in Fe. For He–V clusters with He density ranging 0–6, the binding energy of He atoms and SIAs to He–V clusters decreases with increasing He density, while the binding energy of vacancies increases with increasing of He density. For regime with He density >6, however, the binding energy of He atoms, interstitials and vacancies turns into a contrary trend in comparison with low He density regime [25]. This conspicuous transition should be ascribed to the kick-out of matrix Fe atom induced by high He density in He–V clusters. When He density is >6 in Fe, He atoms exhibit close-packed configurations and exert pressure large enough to produce Frenkel pairs. The kick-out mechanism creates additional vacancies and interstitials, lowering He density in He–V clusters. As shown in Figure 3 [25], the binding energy of He atoms to He–V clusters with low He density is quite high, which indicates that He–V clusters with low He density are strong sinks for He atoms. Therefore, the kick-out mechanism significantly enhances He bubble nucleation and growth.

**Figure 4:**
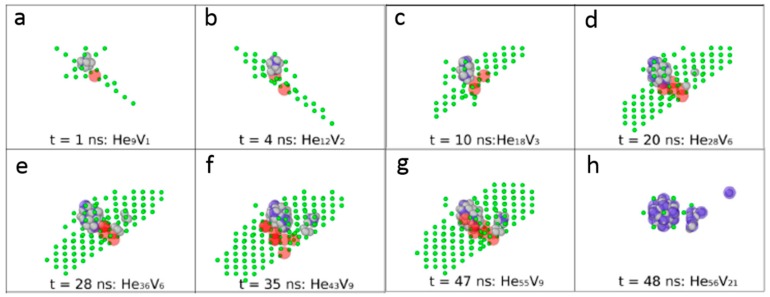
The evolution process of a bubble network at 1000 K with an insertion rate of 1 × 10^9^ He/s in tungsten. (**a**) Formation of the first Frenkel pair and He_9_V_1_ cluster; (**b**) Another Frenkel pair is formed with further inserted of He atoms with two interstitials staying in two adjacent <111> rows; (**c,d**) More Frenkel pairs are created around the central He–V cluster; (**e**) New He cluster containing three He atoms is formed; (**f,g**) He clusters promote Frenkel pairs formation. (**h**) He bubbles in the matrix. Gray, red and blue dots represent He atoms, tungsten interstitials and vacancies, respectively. Reprinted with permission from [33]; Copyright 2018 Elsevier.

Sandoval et al. [33] investigated He bubble nucleation and growth accompanied by kick-out mechanism in detail. Figure 4 schematically illustrates the process of He bubble nucleation and growth accompanied by formation of Frenkel pairs in tungsten. The gray, red and blue spheres represents He atoms, interstitials and vacancies, respectively. In addition, green spheres indicate the deformed tungsten lattice around interstitials. At the very beginning, a cluster containing 8 He atoms is deliberately placed at the center of a tungsten sphere containing 23,538 tungsten atoms. He atoms are implanted at a constant rate (1 × 10^19^ He/s) at 1000 K, which is slow enough to allow the inserted He clusters to diffuse in tungsten matrix and interact with the pre-existing central He bubble [33]. As shown in Figure 4a, the inserted He atoms attach to the pre-existing He cluster, leading to the formation of Frenkel pair and creating a He_9_V cluster. Another Frenkel pair is created with further inserting He atoms in tungsten, as shown in Figure 4b. Notably, two interstitials diffuse around He–V cluster in a coordinated way, staying in two adjacent <111> rows, which is consistent with interstitials arrangement in Fe [27]. Gao et al. [31] proposed that, before forming crowdion along <111> direction, interstitials may create stable configurations with the formation of a <110> dumbbell in Fe, which is consistent with Xiao’s work [37]. In addition, a transition from <100> interstitial cluster to <111> interstitial cluster is identified in tungsten [29,49]. This process is energetically favorable with an energy release of 1.12 eV, indicating that the <111> SIAs cluster is more stable. However, similar orientation transition is not observed in Figure 4. With further He insertion, more Frenkel pairs are created around the central He–V cluster, as shown in Figure 4c,d. Notably, the orientation of SIAs at this stage is kept constant during the following simulation timescales. In addition, the interaction of inserted He atoms with interstitial configurations can be identified clearly. These inserted He atoms can be trapped by interstitials. At 1000 K, these new He atoms attached to interstitials are mobile and can diffuse along interstitial configurations or jump to the He–V cluster. With further increasing of He concentration, new He cluster containing three He atoms is formed near the central He–V cluster, as shown in Figure 4e. A new He cluster can further trigger the formation of Frenkel pairs and act as an embryo for new He bubbles, as shown in Figure 4f,g. These He–V clusters further trap the subsequently inserted He, producing more Frenkel pairs and forming He bubbles in tungsten matrix. Finally, SIAs detach from the initial He bubbles and three helium bubbles are nucleated in the matrix.

The number of He atoms in He clusters needed to create Frenkel pairs has been evaluated using MD simulations and first principle calculations under various conditions. The minimum number of He atoms needed to create Frenkel pairs in Fe is slightly different, which may be attributed to various simulation conditions. For instance, when the Fe matrix is fully dense and without vacancy, three He atoms are believed to be enough to push out matrix Fe atoms [31]. If there are pre-existing vacancies in the simulation box, six He atoms are needed to create Frenkel pairs [25]. The pre-existing vacancies may release the pressure of He clusters, and more He atoms are required to create Frenkel pairs.

### 2.3. Dislocation Loop Punching Mechanism

In Section 2.2, we summarize studies on kick-out mechanism that is commonly observed in He-containing metals. These SIAs pushed out by high pressure He bubbles deliver limited mobility and form crowdion structure around He bubbles. The crowdion structure is observed to be temporarily trapped by He bubbles once formed [31,32,33,34,37,48,49]. Notably, the crowdion structure usually exhibits an energetically favorable orientation along <111> direction in BCC metals [27,29,31,37,49]. With further increasing He concentration, SIAs accumulate continuously around He bubbles, giving rise to the formation and emission of dislocation loops. This well-known growing mechanism of He bubble, named as dislocation loop punching, is widely identified in various metals [27,29,49,50,51,52,53,54,55,56].

**Figure 5:**
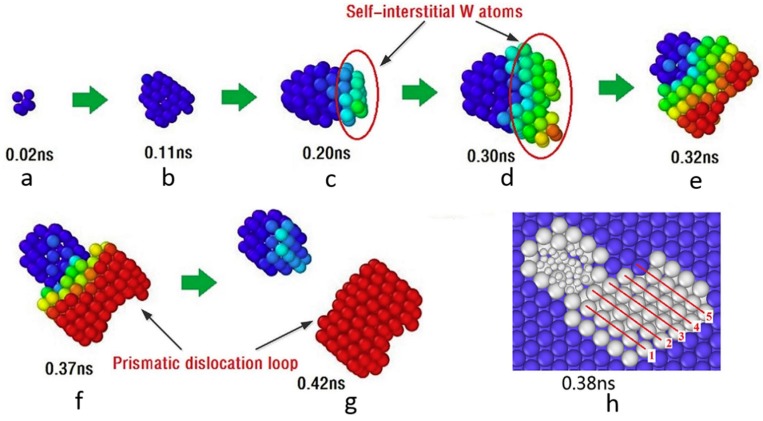
The evolution of He bubble with an initial radius of 0.15 nm. The color from blue to red qualitatively designates the distance from the present atomic site to the center of the He bubble. (**a**,**b**) Homogeneous bubble nucleation and growth; (**c**,**d**) self-interstitial tungsten atoms emit from matrix and then attach to the bubble surface; (**e**–**g**) self-interstitial tungsten atoms evolve into a prismatic dislocation loop and then dissociate from the bubble; and (**h**) the atomic structure of the self-interstitial atom cluster. Reprinted with permission from [49]; Copyright 2017 Elsevier.

Xie and coworkers [49] performed MD simulations to investigate the loop-punching mechanism for He bubble growth in tungsten at 300 K. Figure 5 illustrates the loop-punching process in He bubble with an initial radius of 0.15 nm (containing 1 He atom). From 0.02 to 0.11 ns, He bubble grows homogeneously without emitting defects. With increasing time, He accumulates continuously in He bubble and SIAs are pushed out due to increasing pressure. These SIAs form crowdion configurations along <100> direction, as labeled in Figure 5c,d. In BCC metals, the <100> orientation crowdion configuration is not stable due to its slightly high energy [5,29,49]. At 0.32 ns, the <100> orientation crowdion configuration reorientates to a more stable <111> orientation, thus releasing the high energy of the configuration [49]. A transition from <100> direction to <111> direction of crowdion configuration is also identified in tungsten recently [29]. The <111> crowdion configuration further absorbs subsequent SIAs and evolves into a prismatic loop at 0.37 ns [49]. Finally, the well-developed prismatic loop dissociates from the He bubble and slips away, leaving a larger He bubble (about 0.55 nm) behind. In addition to the conventional loop-punching mechanism, Xie et al. [49] also proposed a new loop-punching mechanism for He bubble with larger initial radius in tungsten. They demonstrated that, for larger He bubbles, the prismatic loop is formed after two screw components cross-slip on the opposite directions. Finally, a dislocation network formed via dislocation interactions surrounds He bubbles (details are shown in Reference [49]).

The kick-out of matrix atoms plays a role of precursor for dislocation loop punching [27,29,49,50,51,52,53,54,55]. SIAs are pushed out first and rearrange their orientations to form dislocation loops. Generally, bubble growth is accompanied by large dislocation loop formation and punching [49,50,51,52,53,54,55]. It is reported that pushing out SIAs and reorientation of SIA cluster can release the high pressure of He bubbles [49]. Dislocation loop punching can only be triggered above certain critical He pressure and also releases He bubble pressure, but less work is conducted on the effect of large loops on He bubbles.

### 2.4. Bubble Coarsening

**Figure 6:**
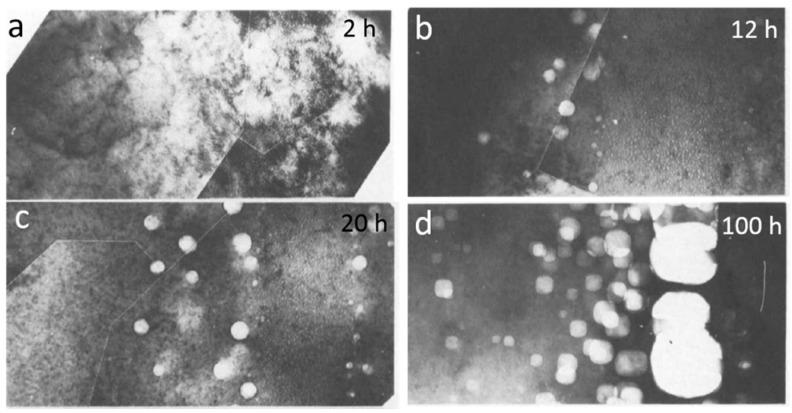
He bubbles in Ni implanted with 1 × 10^17^ ions cm^−2^ at 500 keV, annealed at 750 °C for: (**a**) 2 h; (**b**) 12 h; (**c**) 20 h; and (**d**) 100 h. He bubbles size increases with increasing annealing time. Reprinted with permission from [57]; Copyright 1987 Elsevier.

Bubble coarsening is commonly observed when metals are annealed at a certain temperature that is higher than the temperature at which bubble nucleation and growth proceed [56,57,58,59]. With constant He dose, bubble coarsening inevitably leads to increased average bubble size and reduced bubble number density [56,57,58,59]. Marochov et al. [57] conducted thermal treatment on nickel (Ni) after they implanted He at a dose of 1 × 10^17^ ions/cm^3^. Figure 6 shows He bubble in irradiated Ni annealed at 750 °C for 2, 12, 20 and 100 h, respectively [57]. By comparing bubble morphologies in Figure 6, it is evident that the average bubble size increases obviously with increasing of annealing time, indicating significant bubble coarsening under annealing [57].

Generally, two typical mechanisms, bubble migration and coalescence (BMC) and Ostwald ripening (OR), are proposed for bubble coarsening under annealing [56,57,58,59,60,61]. BMC mechanism mainly depends on the rearrangement of bubble surface through diffusion of internal surface atom [58,59]. Marochov et al. [57] found that small bubbles are preserved for a long time in irradiated Ni under annealing. This seems to be counterintuitive as small bubbles are believed to deliver relatively high mobility. This phenomenon can be rationalized with bubble pressure. Small bubbles usually exhibit high equilibrium pressure due to the high He/V ratio in bubbles [28,57]. The high equilibrium pressure significantly suppresses the diffusion of internal surface atom, which reduces the mobility of small bubbles [57]. More vacancies are required for small bubbles before they coarsen through BMC. In addition, large bubbles develop energetically favorable facets and the mobility is controlled by ledge nucleation [57]. OR mechanism is driven by different equilibrium pressure of bubbles with different sizes [60,61]. This process is controlled by thermally activated dissociation of He–V from one bubble and recombination of He/V to another bubble [56,60,61]. Generally, He–V tends to dissolute from small bubbles and then recombines with large bubbles, leading to shrinkage or even disappearance of small bubbles and coarsening of large bubbles. Obviously, the activation energy for OR mechanism is determined by the energy for dissociation of He–V from bubbles, which is much higher than that for MC mechanism [56]. Therefore, temperature is believed to play a key role in determining the bubble coarsening mechanism under annealing.

Other studies demonstrated that microstructural characteristics (i.e., alloying elements, precipitates, defects etc.) may exert non-negligible effects on bubble behaviors, leading to different bubble coarsening mechanisms. Ono et al. [62] observed bubble migration and coalescence in He-irradiated Fe under 750 °C annealing treatment, while bubbles in Fe-9Cr show much lower mobility. In addition, Roldán et al. [63] conducted post-irradiation annealing experiments on He-irradiated two reduced activation ferritic martensitic steels (EU-ODS EUROFER and EUROFER97). Both steels contain complex microstructures that serve as barriers for bubbles. Large He bubbles are formed in EU-ODS EUROFER, while high-density small He bubbles are observed in EUROFER97. They proposed that different He bubble morphologies in samples should be attributed to different microstructures. Neither OR nor BMC fits perfectly with bubble coarsening under annealing treatment in their samples. These studies indicate that microstructures also significantly influence bubble coarsening mechanism.

## 3. Helium Bubble in Nanocrystalline Metals and Metallic Multilayers

In the preceding section, we overview He bubbles nucleation, growth and coarsening in the interior of grains. Interfaces, mainly GBs and heterophase interfaces (bi-metal interfaces), have attracted tremendous interests in the past decades [64,65,66,67,68,69,70,71,72,73,74,75,76,77,78,79,80,81,82,83,84,85,86,87,88,89,90,91,92,93,94,95,96,97,98,99,100,101,102,103,104,105,106,107,108,109,110,111,112]. Two reasons are responsible for the tremendous research interest of interfaces. Firstly, He bubbles at GBs are most harmful due to their tendency of coalescence under stress or at elevated temperature. In addition, a transition from bubble to void is widely identified in metals containing bubbles [64,65,66,67]. At certain temperature, bubbles larger than a critical size evolve into voids by absorbing vacancies quickly [64,65,66,67]. For GB He bubbles, this transition is particularly deleterious and always lead to high temperature helium embrittlement in metals [67]. More details are discussed in Section 5. Secondly, GBs and heterophase interfaces are sinks for radiation-induced defects, which are usually used to design radiation tolerant materials [64,68,69]. Serving as efficient sinks for radiation defects, interfaces can mitigate and recover various defects produced in metals after high displacement damage [68,69]. Singh [70] found that austenitic stainless steel (SS) exhibits enhanced radiation tolerance with reducing grain sizes as GBs serve as sinks for radiation defects. Similarly, Zhang et al. [23] reported that He bubble size and density all decrease with increasing interface density in He irradiated Cu/V multilayers. Using three atomistic simulation methods, Bai et al. [68] proposed a novel “loading-unloading” mechanism to rationalize the strong sink effect of GBs in Cu. They recognized that interstitials are loaded into GBs under irradiation and then are emitted to annihilate vacancies in bulk [68]. Ackland [69] pointed out that the “loading-unloading” mechanism is general and may also work at heterophase interfaces. Therefore, GBs and heterophase interfaces are introduced into metals deliberately to enhance radiation tolerance [64]. Generally, nanocrystalline (NC) metals have higher radiation resistance than their coarse-grained (CG) counterparts, and nanolaminates show enhanced radiation tolerance than their bulk single-phase counterparts. In this section, we introduce the influence of interface on He bubbles agglomeration in metals. A fundamental understanding on bubble–interface interactions is crucial for managing He bubbles, mitigating He-induced degradation of interface and designing of radiation-tolerant metals.

### 3.1. Helium Bubbles in Nanocrystalline Metals

**Figure 7:**
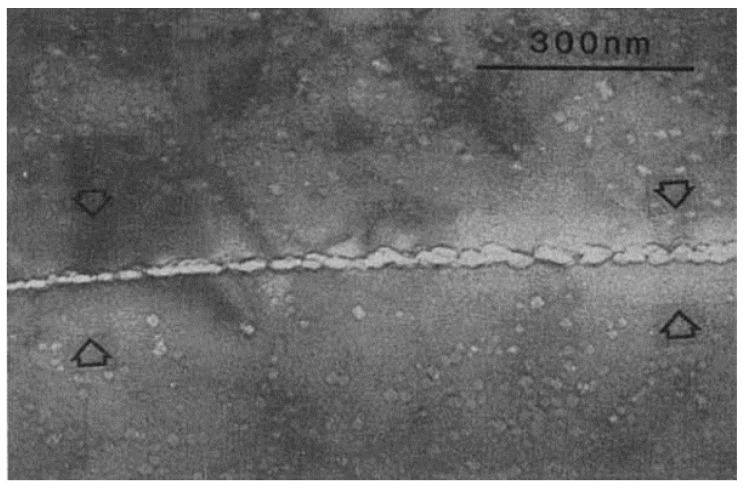
Large He bubbles along GB in He irradiated austenitic steel. As indicated by arrows, BDZs with width of tens of nanometers distribute symmetrically on both sides of GB. Reprinted with permission from [74]; Copyright 1983 Informa UK Limited.

GBs, as common defects in polycrystalline metals, have sharply different atomistic structures from that of interior of grains. Sun et al. [71] performed in situ Kr irradiation on both CG and NC Ni and found that high-density GBs in NC Ni significantly reduce density and size of irradiation induced defects. Similarly, some other investigations [72,73] confirm that NC metals deliver enhanced radiation resistance compared with CG counterparts. Both experiments [74,75,76] and simulations [77,78,79] report that He atoms tend to segregate to GBs and form He clusters/bubbles along GBs. Due to the sink effect of GB [79], this process is activated under irradiation and post-annealing at various temperatures [74,75,76,77,78,79]. A vivid example for He precipitation along GBs is shown in austenitic steel (Figure 7) [74]. Notably, He bubbles formed along GB are larger than bubbles located in the interior of grains. As marked by arrows in Figure 7, bubble-denuded zone (BDZ), with a width of tens of nanometers, is observed on both sides of GB [74]. BDZs, similar to void-denuded zones in Cu [80,81], indicate that GB may absorbs adjacent He and vacancies and produces poor-He zones on both sides. Similarly, approximately 10 nm-wide BDZs are identified in He-implanted ferritic alloy [75]. Taking Fe as model material, Kurtz et al. [79] evaluated the trapping efficiency of He at substitutional and interstitial sites in and near GBs. They found that interstitial He atom was strongly binding to GB core with an energy of 0.5–2.7 eV [79]. Surprisingly, even at 0 K, GBs still serve as activated He sinks and deliver a He capture radius ranges from 0.3 to 0.7 nm [79]. Considering the high mobility of He atoms in metals [27,31,64], the effective capture radius of GBs may be 1–2 orders of magnitude higher than that of calculated here. This indicates that the effective capture radius may reach several nanometers, even tens of nanometers. This simulation rationalizes the formation of BDZs in He irradiated austenitic steel [74] and ferritic alloys [75]. Series of complex factors, including intrinsic factors (matrix, GB misorientations [79,80], GB energies [80], etc.) and extrinsic factors (temperature [82], He concentration [64], etc.), are supposed to exert a non-negligible effect on BDZs formation and evolution.

**Figure 8:**
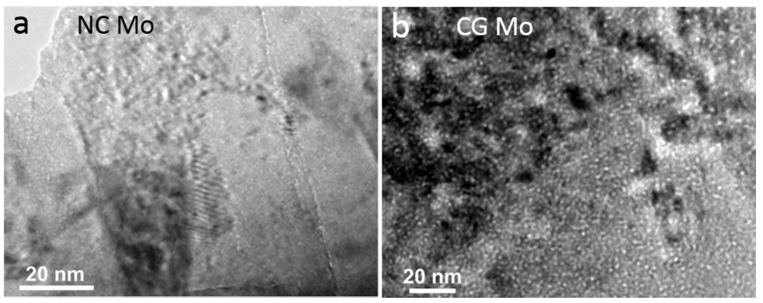
He bubbles induced by He irradiation in: NC Mo (**a**); and CG Mo (**b**) [83]. Small He bubbles with an average size of 0.6 nm are observed in NC Mo, while large He bubbles with an average size of 1.2 nm are identified in CG Mo. Reprinted with permission from [83]; Copyright 2016 Elsevier.

Many studies [82,83,84,85] are performed to unveil the dependence of radiation tolerance on grain size and explore the underlying mechanisms on both CG metals and NC metals. Due to their huge disparity in the ratio of GB area to total volume, CG metals and NC metals irradiated by He at the same condition deliver quite different bubble morphologies and distributions [82,83,84,85]. For He irradiation, the enhanced radiation tolerance in NC metals is usually manifested as small and low-density bubbles [82,83,84,85]. A representative example for He precipitation in NC Mo and CG Mo is shown in Figure 8 [83]. In NC Mo with an average grain size of 44 nm, small and sparse bubbles are formed after He irradiation (Figure 8a). The average bubble size is about 0.6 nm. The CG Mo are decorated with dense bubbles with an average size of about 1.2 nm. This finding is also confirmed in NC Fe and CG Fe. Yu et al. [84] proposed that smaller and lower-density bubbles in NC metals should be attributed to the depletion of vacancies from grain interior induced by high-density GBs. As mentioned above, Bai et al. [68] reported that GBs can capture interstitials under irradiation, and then play a role of interstitial emitter and fire interstitials back into the lattice to recombine with vacancies that stay within a few nanometers to GBs (recombination area). NC metals possess a rather high ratio of GB area to total volume, which means many traps for interstitials are produced in collision cascade. More importantly, with reducing grain size, the ratio of width of recombination area to grain size will generally reach 1, which indicates that the overall vacancies in the interior of grains have a great chance to recombine with interstitials emitted by GBs, leading to the low concentration of vacancies [68,84]. The depletion of vacancies gives rise to smaller and lower-density of He bubbles in NC grains.

Bullough et al. [86] evaluated the dependence of sink strength on grain size. Using the cellular model, they concluded that the sink strength for GB can be describe as

(1) $$k_{\mathrm{gb}}{}^{2}=15/R^{2}$$

where $k_{\mathrm{gb}}{}^{2}$ is the GB sink strength and *R* is grain size. The cellular model focuses on a single spherical isolated grain without identified surrounding medium and uses the average point defect concentration within the grain. When the embedding model is used, the sink strength of GB can be calculated as

(2) $$k_{\mathrm{gb}}{}^{2}=14.4/R^{2}$$

where $k_{\mathrm{gb}}{}^{2}$ and *R* have the same meaning as in Equation (1). It is evident that sink strength of GB predicted using both models delivers similar values. This result unveils the trend that the sink strength increases as the grain size decreases. However, it should be noted that there are some limits for these two formulas, although they predict the dependence of sink strength on grain size [87]. Firstly, according to these formulas, the sink strength can become larger and larger as the grain size decreases. This seems to be unreasonable [87]. Secondly, these formulas ignore the dependence of sink strength on GB characters. For instance, high angle GBs (HAGBs) usually possess higher sink strength compared to low angle GBs (LAGBs), as HAGBs deliver lower formation energy for interstitials and vacancies [79,80,87]. Thirdly, the sink strength of GBs is reduced after absorbing tremendous point defects [87]. In addition, grain growth in NC metals is also inevitable under irradiation, especially at high temperature [113]. Considering these issues listed above, an alterable factor that determined by GB energy (γ) and GB orientation (θ) should be involved. The sink strength of GB can be calculated using a modified formula [87]

(3) $$k_{\mathrm{gb}}{}^{2}=15f\left(\theta ,\gamma \right)/R^{2}$$

Generally, the *f*(θ,γ) ranges from 0 to 1. Further effort is needed to investigate the dependence of *f*(θ,γ) on GB energy (γ) and GB orientation (θ), which is out of the scope of this review.

### 3.2. Helium Bubbles in Metallic Multilayers

Tremendous studies [74,75,76,77,78,79,80,81,82,83,84,85] have demonstrated that NC metals deliver dramatically enhanced radiation tolerance compared with their CG counterparts. However, NC metals are not widely adopted as candidate structural materials in nuclear reactors that are subjected to a high level of irradiation because of their thermal instability [113]. Generally, for NC metals containing grains with size of about 10 nm, an arresting grain growth at room temperature or even below can be identified if the equilibrium melting temperature is lower than about 873 K [113]. The temperature triggering grain growth rises with increasing grain size and melting temperature, but is still not high enough compared with that of real service environment.

Interface engineering is regarded as an important method to achieve high radiation resistance in metals [88,89,90,91,92,93,94,95,96,97,98,99,100,101,102,103,104,105,106,107,108,109,110,111,112]. In fact, interface engineering shares the same fundamental principle with NC metals. That is, in short, increasing the ratio of interface area to total volume enhances the radiation tolerance. As interfaces serve as efficient defect sinks, recombination of irradiation-induced point defects, mainly interstitials and vacancies, is promoted with increasing interface density, hence giving rise to enhanced radiation tolerance [88,89,90,91,92,93,94,95,96,97,98,99,100,101,102,103,104,105,106,107,108,109,110,111,112]. Radiation resistant multilayers consisting of alternative nanoscale layers are designed by introducing high-density heterophase interfaces and exhibit much better radiation tolerance than either of its single-phase components [94,107,108]. The evolution of radiation defects, including dislocation loops, He bubbles, voids etc., are dramatically suppressed by the high-density of heterophase interfaces [88,89,90,91,92,93,94,95,96,97,98,99,100,101,102,103,104,105,106,107,108,109,110,111,112]. Yu et al. [111] provided in situ observation of the phenomenon that heterophase interfaces capture and annihilate Kr irradiation-induced defect clusters in immiscible Ag/Ni multilayers. 

Design of multilayers containing high-density heterophase interfaces to alleviate He irradiation damage is based on the theory that interfaces can trap He and vacancies efficiently and provide abundant nucleation sites for He bubbles [64]. Studies on He-irradiated multilayers demonstrated that heterophase interfaces play a critical role in improving radiation tolerance. Li et al. [93] characterized He bubble size and distribution in Cu and 5 nm Cu/Nb multilayers after 7 at.% He irradiation. They found that the average bubble radius in Cu/Nb multilayers is about 0.5 nm, which is much smaller than bubbles in Cu (1.35 nm). In addition, the bubble volume fraction in Cu/Nb multilayers is only about 1.74%, while the bubble volume fraction in Cu reaches 5.77%. Similarly, compared to Ag, He bubbles in 6 nm V/Ag multilayers are smaller in both size and volume friction after He irradiation [105]. VDZs [80,81] and BDZs [108] are also identified in multilayers, suggesting the critical role of heterophase interface in suppressing irradiation defects. In past decades, there are many successful examples in the design of multilayers containing high-density heterophase interfaces to achieve outstanding radiation tolerance and mechanical properties. These studies are mainly dedicated to radiation behaviors in a series of multilayers, including Cu/Nb [80,88,89,90,91,92,93,94,95,96,97,112], Cu/V [98,99,100], Cu/Mo [101], Cu/Co [102],Cu/W [103], Fe/W [104], V/Ag [105,106], Al/Nb [107], Cu/Ag [108,109] and Ag/Ni [110,111]. Among them, semi-coherent immiscible FCC/BCC heterophase interfaces [32,88,89,90,91,92,93,94,95,96,97,103,105,106,107] have been investigated both experimentally and theoretically, and this review focuses on this system. 

**Figure 9:**
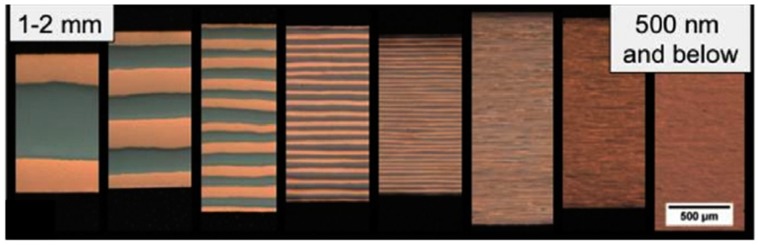
Multilayers manufactured using ARB with layer thickness ranging from millimeters to nanometers. Reprinted with permission from [64]; Copyright 2015 Elsevier; Reprinted with permission from [112]; Copyright 2013 Cambridge University Press.

Different laboratory methods have been developed to manufacture multilayers with various layer thickness, such as magnetron sputtering [92,93] and accumulated roll bonding (ARB) [80]. By tuning manufacture parameters, multilayers with various layer thickness can be obtained for experimental investigations. Figure 9 schematically illustrates the manufactured Cu/Nb multilayers by using ARB method [64,112]. The original bulk Cu and Nb plates are stacked together and then processed by continuous rolling, and repeated cutting and stacking; as a result, Cu/Nb multilayers with layer thickness ranging from millimeters to nanometers can be produced. Figure 9 [64,112] shows typical layered microstructures formed in ARB Cu/Nb multilayers. The Cu/Nb interfaces are planar and sharp. The controllable layer thickness makes it easy to tune the density of heterophase interface, which provides an opportunity to evaluate the dependence of radiation tolerance on the density of heterophase interface. Notably, novel interfaces are found in multilayers processed using ARB. In ARB Cu/Nb multilayers, {112}_FCC_||{112}_BCC_ and {110}_FCC_||{112}_BCC_ are two of the dominant interface orientations [64,112]. Contrary to ARB, FCC/BCC multilayers synthesized using magnetron sputtering usually have similar interface orientation relationship (except for V/Ag multilayers [90,105,106]). For instance, all heterophase interfaces in Cu/Nb magnetron sputtered multilayers have the Kurdjumov–Sachs orientation relationship with interface plane of {111}_FCC_||{110}_BCC_ [89,90,91,92,93,94,95,96,97]. 

**Figure 10:**
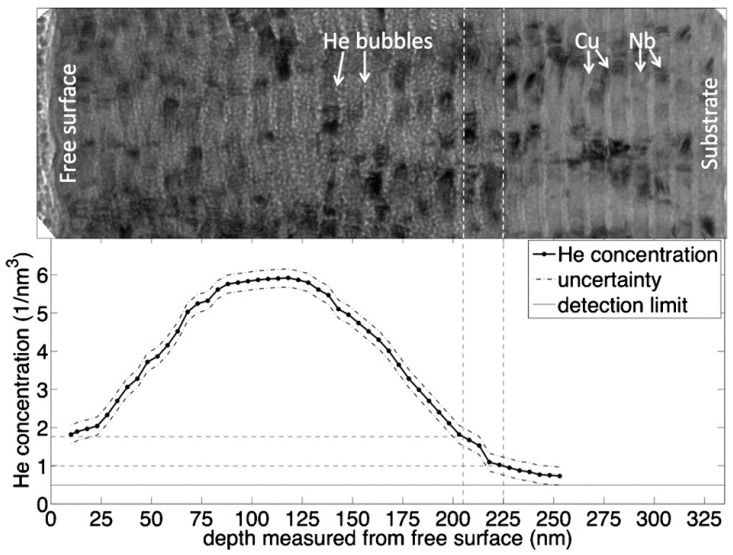
TEM image of He^3^-implanted Cu–Nb multilayers with layer thickness of 5.6 nm and He concentration profile measured using NRA. For region below a critical depth around 215 nm, no He bubble can be observed. Reprinted with permission from [90]; Copyright 2010 AIP.

As mentioned above, He has extremely low solubility in metals. He atoms, once introduced into metals, are supposed to precipitate and form bubbles quickly. Surprisingly, Demkowicz et al. [90] found that no He bubbles were identified in region with low but not zero He concentration via TEM in magnetron sputtered Cu/Nb multilayers after He^3^ irradiation [90]. In Figure 10, the upper image shows Cu/Nb multilayers after He^3^ irradiation, while the lower figure describes the evolution of He^3^ concentration along depth. The He^3^ concentration is measured by nuclear reaction analysis (NRA), instead of estimation by using the Stopping and Range of Ions in Matter (SRIM) [114], as NRA accurately reflects the true He^3^ depth profile [90]. He bubbles are only observed at high He concentration region, while no He bubbles are detected in low He concentration region. Based on this sharp comparison, it is inferred that small He clusters, which are too small to be detected by TEM, are captured by Cu/Nb heterophase interfaces in low He concentration region. With increasing He concentration, these small clusters evolve into visible bubbles, as shown in the middle part of Figure 10. This comparison indicates that there exists a critical He concentration to form visible He bubbles (under TEM) along interfaces. 

To determine the critical He concentration to form visible He bubbles in Cu/Nb multilayers, Demkowicz et al. [90] performed He^3^ irradiation on several multilayers with different layer thicknesses. These irradiated multilayers are characterized using TEM with under-focus mode to detect He bubbles. He^3^ concentration as a function of depth is evaluated using NRA. When converted to number of He atoms per unit of interface area (rather than sample volume), the critical He concentration for formation of visible He bubbles is about 8.5 atoms/nm^2^ in Cu/Nb multilayer, which is irrelevant to layer thickness [90]. Analogous studies find that the critical He concentrations to form visible He bubbles in Cu/V multilayers [98,99,100] and Cu/Mo [101] multilayers are 1.9 atoms/nm^2^ and 3 atoms/nm^2^, respectively. The sharply different critical He concentrations in various systems are ascribed to different areal densities of misfit dislocation intersections (MDIs) in various heterophase interfaces [90,98,99,100,101]. 

**Figure 11:**
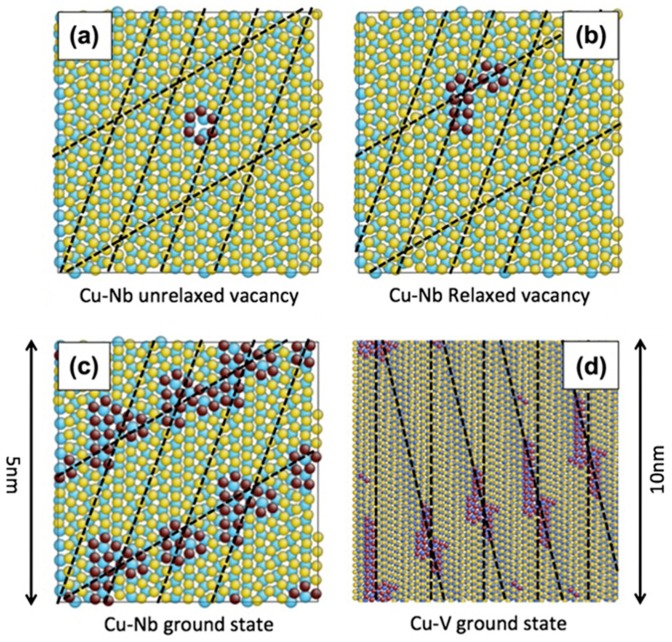
Unrelaxed (**a**) and relaxed (**b**) vacancies on the Cu side of a Cu–Nb interface; (**c**) about 5 at.% constitutional vacancies locates in the ground state Cu–Nb interface; and (**d**) about 0.8 at.% constitutional vacancies locates in the ground state Cu–V interface. Vacancies tend to gather around MDIs. Reprinted with permission from [95]; Copyright 2012 Elsevier.

Atomistic modeling revealed that {111}_FCC_||{110}_BCC_ interfaces contain two sets of parallel misfit dislocations [95], forming a series of MDIs on interfaces. As demonstrated using atomistic modeling in Reference [95], these MDIs are preferred He bubble nucleation sites. Figure 11a shows the effect of creating a vacancy near a misfit dislocation in Cu plane. After annealing for 10 ps at 300 K followed by energy minimization, the vacancy occupies the MDI with a low formation energy of −0.13 eV. This configuration indicates that interface decorated by constitutional vacancy is energetically favorable to the vacancy-free interface. Constitutional vacancies were continuously added to the heterophase interface until no negative formation energy sites remain. The final Cu/Nb interface containing about 5 at.% constitutional vacancies gathered near MDIs has about 25 mJ/m^2^ lower energy than a perfect interface. That is, about 2.5 constitutional vacancies gather near one MDI (Figure 11c). Similarly, a Cu/V interface with 2.5 constitutional vacancies on single MDI (Figure 11d) and an energy reduction of 3.4 mJ/m^2^ is formed finally. It should be noted that, due to different lattice parameters, the areal density of MDIs on Cu/Nb interface is over a factor of five larger than that on Cu/V interface [90,98,99,100]. This results in the atomic percentage of constitutional vacancies (about 0.8 at.%) on Cu/V interface being much smaller than that on Cu/Nb interface (about 5 at.%) [90]. Due to the high binding energy, He atoms are easily trapped by constitutional vacancies locating on MDIs and form He–V clusters. These He–V clusters can be regarded as He bubble embryo and may develop into He bubbles. Hence, MDIs are preferred He bubble nucleation sites. Notably, the amount of He atoms trapped by a MDI should be constant before forming a visible He bubble. The areal density of MDIs is five times higher for Cu/Nb interface than that for Cu/V interface, which corresponds very well with the fact that the critical He concentration to form visible He bubbles on Cu/Nb interface (about 8.5 atoms/nm^2^) is also about five times higher than that on Cu/V interface (about 1.9 atoms/nm^2^). This attests that different areal densities of MDIs are responsible for various critical He concentration in different multilayers.

**Figure 12:**
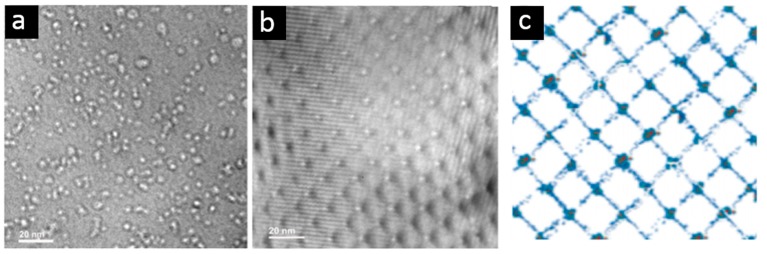
(**a**) Dense He bubbles in single-crystal Au after He irradiation; (**b**) He bubbles distribute on MDIs in a (001) twist grain boundary with a misorientation angle about 1°; and (**c**) Metropolis Monte Carlo simulation shows dislocation network in blue and He bubble at MDIs in red. Reprinted with permission from [115]; Copyright 2012 Elsevier.

MDIs in heterophase interfaces are efficient sinks for He. This deduction is further proved in bi-crystal Au film, as shown in Figure 12 [115]. He implantation is conducted on both single-crystal and bi-crystal Au foil containing a twist boundary. Dense He bubbles with various bubble size are homogeneously distributed in single-crystal Au foil, as shown in Figure 12b. Interestingly, He bubbles are observed to array periodically on dislocation intersections in bi-crystal Au. The twist boundary comprises a square grid of screw dislocations [115]. All dislocation intersections are occupied by He bubbles, while no bubbles are identified in matrix. Similar bubble distribution on dislocation intersections is further demonstrated using Metropolis Monte Carlo simulation (Figure 12c) [115]. From the comparison between single-crystal Au and bi-crystal Au, it is evident that dislocation intersections are preferred He bubble nucleation sites. Similarly, MDIs in heterophase interfaces decorated with vacancies can trap He efficiently [95].

**Figure 13:**
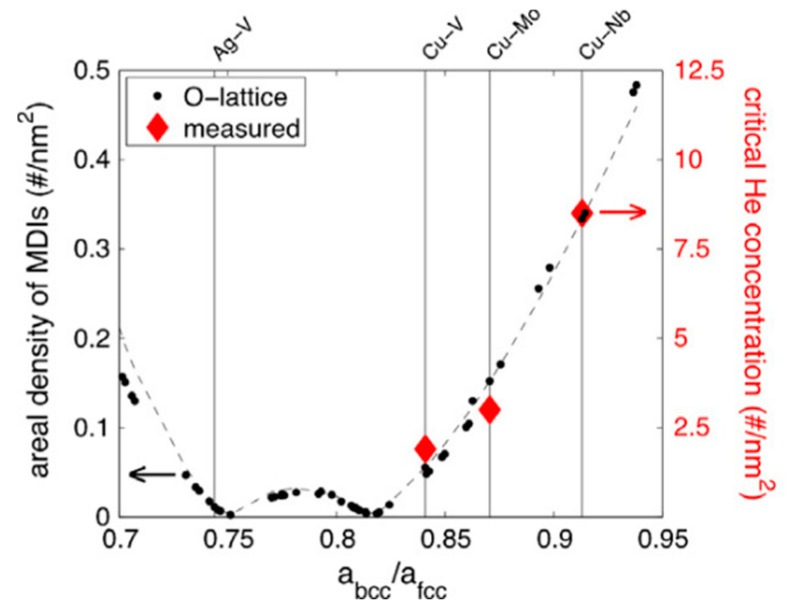
Black dots show the dependence of areal densities of MDIs calculated using O-lattice theory on lattice parameter ratios in FCC/BCC interfaces with identical interface orientation relationship. Red diamonds represent the critical He concentration to form visible He bubbles with different lattice parameter ratios. Reprinted with permission from [95]; Copyright 2012 Elsevier.

Figure 13 [95] plots the areal density of MDIs and the critical He concentration to form visible bubbles as a function of the lattice parameters ratio. The areal density of MDIs in FCC/BCC heterophase interfaces can be evaluated using O-lattice theory, which is described in details in Ref. [116]. The areal density of MDIs in Ag/V, Cu/V, Cu/Mo and Cu/Nb interfaces calculated adopting O-lattice theory are plotted in Figure 13 [95]. Generally, the MDI areal densities vary with different lattice parameter ratio without a clear trend for ratio ranging from 0.7 to 0.95. However, for lattice parameter ratio ranges from about 0.82 to 0.95, the areal density of MDIs calculated using O-lattice theory increases monotonically with increasing lattice parameter ratio (Figure 13). The Cu/Nb interfaces have the largest lattice parameter ratio and highest areal density of MDIs. Similarly, the measured critical He concentration also increases monotonically with increasing lattice parameter ratio, as shown in Figure 13. The dependence of critical concentrations on lattice parameter ratios matches well with that of the MDI areal densities. This further attests that various critical He concentrations should be ascribed to different areal densities of MDIs in FCC/BCC multilayers. Based on the areal densities of MDIs and the critical concentrations, it can be determined that about 25 He atoms can be stored on each MDI without forming a visible He bubble in Cu/Nb, Cu/V and Cu/Mo multilayers [90]. According to Figure 13, it seems that Ag/V multilayers contain extremely low-density of MDIs. This should be ascribed to the fact that neighboring Ag and V layers in magnetron sputtered Ag/V multilayers deliver a variety of orientation relations, including Kurdjumov–Sachs, Nishiyama–Wasserman, Bain, and Pitsch orientation relations [105]. Moreover, each successive layer in Ag/V multilayers is polycrystalline. These heterogeneous structures in Ag/V multilayers do not mean that Ag/V multilayers have lower radiation tolerance compared to other FCC/BCC multilayers. In fact, Ag/V multilayers exhibit similar He-irradiation tolerance to Cu/Nb multilayers [90,105].

Heterophase interfaces in semi-coherent FCC/BCC multilayers show unprecedented He storage capacity, which is far beyond the equilibrium solubility of He in metals. The phenomenon that one MDI can sustains 25 He atoms without forming visible bubbles is not unexpected and attracted tremendous interests [88,89,90,91,92,93,94,95,96,97,98,99,100,101,102,103,104,105,106,107,108,109,110,111]. Recent atomistic modeling work focused on He atom behaviors at MDIs rationalized why one MDI can sustain so many He atoms without forming visible bubbles. A He cluster evolution process, briefly described as “platelet to bubble” transition, is proposed [96] to explain the unprecedented He storage capacity of interfaces. 

**Figure 14:**
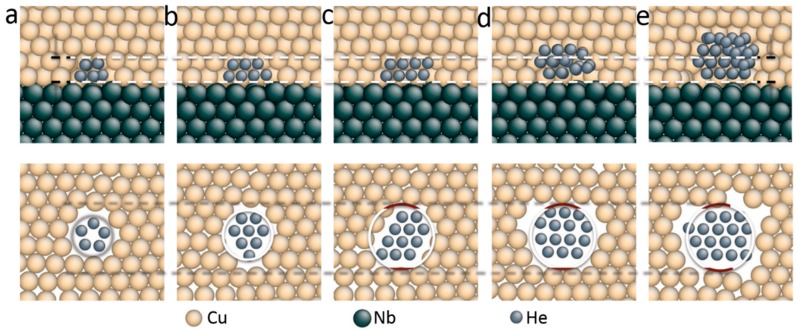
The evolution process of He cluster along Cu/Nb interface: (**a**–**c**) He platelet is formed by wetting high-energy interface regions and extends along the interface; and (**d**,**e**) He platelet transforms into He bubble by adding a new layer of He and keeps its area along the interface constant. Reprinted with permission from [96]; Copyright 2013 NLM.

He bubbles in crystalline metals tend to evolve into stable spherical bubbles due to high isotropic equilibrium pressure. However, low concentration He may form stable He platelets at interfaces [96]. As shown in Figure 14a–c, He platelet is formed by wetting high-energy interface regions and extends along the interface with increasing He concentration in Cu/Nb multilayers. The shape and location of the He platelet is determined by three surface energies. These surface energies are the Cu/He surface energy $\gamma_{\mathrm{Cu}/\mathrm{He}}$, the Nb/He surface energy $\gamma_{\mathrm{Nb}/\mathrm{He}}$ and the Cu/Nb interface energy $\gamma_{\mathrm{Cu}/\mathrm{Nb}}$. An excess interface wetting energy is then described as [96],

(4) $$W=\gamma_{\mathrm{Cu}/\mathrm{Nb}}+\gamma_{\mathrm{Cu}/\mathrm{He}}-\gamma_{\mathrm{Cu}/\mathrm{Nb}}$$

when W>0, the He platelet is expected to wet the interface at a contact angel that depends on the surface energies to minimize the total energy. The surface energies $\gamma_{\mathrm{Cu}/\mathrm{He}}$ and $\gamma_{\mathrm{Nb}/\mathrm{He}}$ are calculated to be 1.93 and 2.40 J/m^2^, respectively. The average interface energy $\gamma_{\mathrm{Cu}/\mathrm{Nb}}$ is 0.54 J/m^2^ [96]. The excess interface wetting energy should always be positive, indicating that, regardless of size, He cluster will always wet the interface in the form of He platelet. However, this hypothesis is valid for He platelet containing fewer than 20 He atoms. This is generally consistent with the former result of 25 He atoms [90]. With further increasing He concentration, the He platelet grows through adding a new layer of He and keeps its area along the interface constant, as shown in Figure 14d. This indicates that a transition from platelet to bubble happens. Further increasing He concentration promotes bubble growth, as shown in Figure 14e.

**Figure 15:**
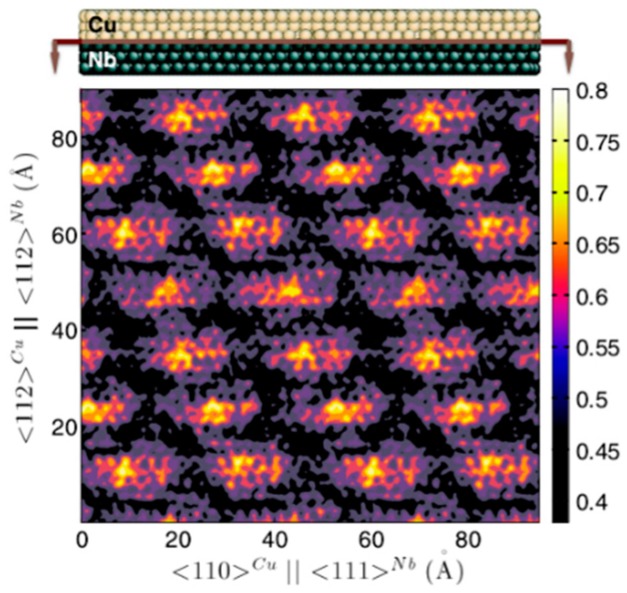
Location dependence of Cu/Nb interface energy. Areas occupied by MDIs deliver the highest interface energy. Reprinted with permission from [96]; Copyright 2013 NLM.

Due to the existence of MDIs in semi-coherent Cu/Nb interfaces, $\gamma_{\mathrm{Cu}/\mathrm{Nb}}$ is not homogeneous across the interface and varies with positions, as shown in Figure 15 [96]. The high energy areas deriving from MDIs in Figure 15 have an interface energy of about 0.8 J/m^2^, while the low energy areas have an interface energy of about 0.4 J/m^2^, leading to an average interface energy of about 0.54 J/m^2^ [90]. The heterogeneity of interface energy across the interface is responsible for the platelet to bubble transition [96]. For high interface energy regions, the excess interface wetting energy *W* is always positive, giving rise to formation of He platelet by wetting the interface. With increasing of He concentration, the He platelet extends by further wetting the interface [96]. However, once the He platelet is large enough and exceeds the original high interface energy region, *W* will be negative, which inhibits further wetting. As a result, the growth of He platelet proceeds into Cu due to the lower vacancy formation energy in Cu. Finally, the platelet transforms into bubble and visible bubbles are detected along interfaces [96].

The above model describes He precipitation along semi-coherent FCC/BCC interfaces innovatively and reasonably. MDIs along interfaces play a critical role in tuning radiation defects and contribute to the high radiation resistance in NC metals and multilayers. MDIs deliver outstanding He storage capacity besides enhancing interstitials and vacancies recombination. An important conclusion derived from above model is that design of interfaces with high-density MDIs is a potential way to achieve enhanced radiation tolerance in materials. These insights provide a practical way to predict He behaviors along interfaces, such as formation of He platelets and bubbles. In addition, the He concentration that a certain multilayer may sustain before forming visible He bubbles along interfaces can be calculated using MDI density. This would surely facilitate the prediction of lift-time of various multilayers exposed to He irradiation. More importantly, these insights guide the design of multilayers and provide new avenues for manufacturing radiation-tolerant materials. However, to apply the interface engineering strategy in real application, a new method that can be scale-up to fabricate interface-dominated metals in industry is still needed.

## 4. Helium Radiation Hardening

In this part, we overview irradiation hardening induced by He bubbles. Most investigations on the strengthening effect of He bubbles are conducted using direct He implantation to introduce a high density of He bubbles within a short range of time [88,89,90,91,92,93,94,95,96,97,98,99,100,101,102,103,104,105,106,107,108,109,110,111]. However, He implantation usually forms He bubbles within a shallow region due to the limitation of ion energy. The depth of He-bubbled region generally ranges from hundreds of nanometers to several micrometers [88,89,90,91,92,93,94,95,96,97,98,99,100,101], which depends on the energies of incident He ions. Hence, nano/micro-mechanical tests are widely adopted to evaluate He bubble induced hardening in these sample. Nanoindentation [63,116,117], and in situ mechanical testing in SEM [118,119,120,121] and TEM [122,123,124,125,126,127] are widely performed to investigate the effect of He bubbles on the mechanical properties of various metals. We mainly focus on three parts next: He irradiation hardening in single-phase metals, He irradiation hardening in metallic multilayers and theoretical modeling of He irradiation hardening.

### 4.1. Helium Radiation Hardening in Single-Phase Metals

**Figure 16:**
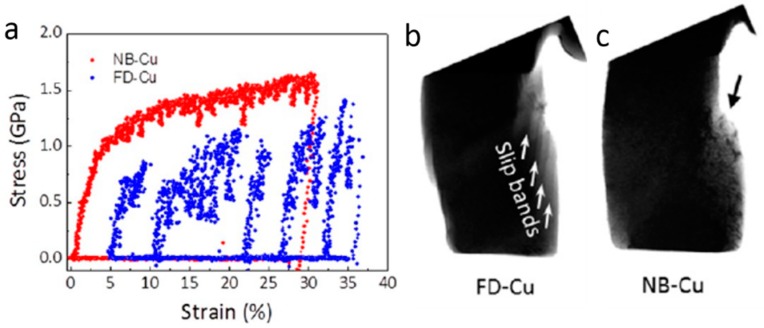
Compression tests on FD-Cu (unirradiated) and NB-Cu (irradiated) pillars: (**a**) NB-Cu containing nanoscale He bubbles deliver stable deformability and higher flow strength; (**b**) sharp slip bands are identified in FD-Cu pillar; and (**c**) smooth deformation profile is identified in NB-Cu pillar. Reprinted with permission from [122]; Copyright 2016 American Chemical Society.

We start the discussion with He irradiation hardening in single-phase metals. Several studies are performed on He irradiated steel [63], Ni [120], Cu [118,122,123,124,125,126] and Al–4Cu alloy [127] to evaluate He radiation hardening and interactions of He bubble with dislocations/deformation twin [63,120,121,122,123,124,125,126,127]. Using nanoindentation teats, Roldan et al. [63] found that He-irradiated steels after annealing demonstrate much higher hardness compared with as-received samples. We [122] demonstrated that He bubbles with an equilibrium internal pressure slightly less than 1 GPa serve as shearable obstacles and internal dislocation sources in single-crystal Cu pillars. As shown in Figure 16a, pillars containing He bubbles deliver higher flow stress and more stable deformation compared with the deformation of fully dense Cu pillars. He bubbles reduce dislocation velocity and mean free path, increase shear stress, and promote the storage of dislocations, giving rise to enhanced yield strength, flow stress and strain hardening in Cu pillars. This is verified by in situ nanomechanical measurement and videos of dislocation–He bubbles interaction in both fully dense and He-bubbled Cu pillars [122]. Sharp slip steps can be identified on surface of deformed fully dense Cu pillar (Figure 16b), while He-bubbled pillars form smooth front surface after large strain of compression (Figure 16c). More importantly, we found that He bubbles can enhance ductility in small-volume Cu pillars [122], which is contrary to the widely accepted He-induced ductility lost in metals [18,19]. The reasons for enhanced ductility are discussed in next section. Similarly, another related study on He irradiated Cu [126] indicates that He bubbles performed as obstacles for both dislocations and twins, leading to enhanced yield strength and flow stress of Cu pillars. In addition, the authors of [120,126] reported a dose-dependent He irradiation hardening in Ni and Cu. The flow stress in both Cu and Ni pillars increases significantly with increasing of He concentration [120,126]. In tensile tests, the ultimate tensile strengths (UTSs) for fully dense single-crystal Ni, and Ni after He implantation with a fluence of 2 × 10^17^ ions cm^−2^ and 3.8 × 10^17^ ions cm^−2^ are 241, 384 and 503 MPa, respectively. The dose-dependent He irradiation hardening should be common in various monolithic metals, but there may exist a critical He concentration over which the He irradiation hardening keeps constant or even decreases with further increasing of He concentration, as demonstrated in He implanted pure Ag [106]. Below the critical He concentration, the spacing of He bubbles reduces continuously with increasing He concentration due to bubble growth and new bubble nucleation. Once the critical He concentration is reached, bubble coalescence, bubble coarsening or bubble-to-void transition may take place, leading to a complex mechanical response. The detailed bubble evolution and their mechanical effects at high He concentration need to be further studied experimentally and theoretically. 

### 4.2. Helium Radiation Hardening in Metallic Multilayers

**Figure 17:**
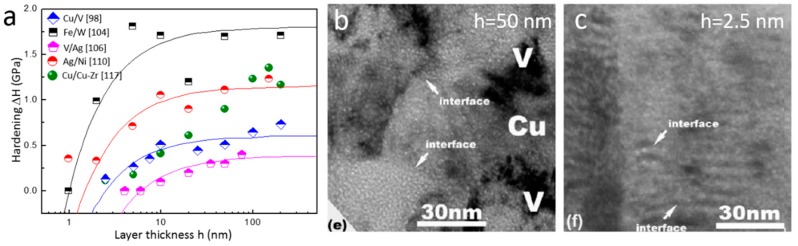
(**a**) Size dependent radiation hardening in various multilayers [98,104,106,110,117]; (**b**) TEM images of He irradiated Cu/V with a layer thickness of 50 nm [100]; and (**c**) He irradiated 2.5 nm Cu/V multilayers [100]. Reprinted with permission from [100]; Copyright 2009 Elsevier.

In this section, we review He radiation hardening in multilayers. Nanoindentation tests on Cu/V multilayers with various layer thickness reveal a size-dependent He bubbled-induced hardening [100]. For layer thickness ranging from 200 to 2.5 nm, the magnitude of He bubble-induced hardening decreases with reducing layer thickness, and the hardening effect is negligible for layer thickness of 2.5 nm or less. Notably, this size-dependent irradiation hardening is also identified in other multilayers, such as V/Ag [106], Ag/Ni [111], Fe/W [104], Cu/Cu–Zr [117], etc. (Figure 17a). Many studies indicate that the size-dependent irradiation hardening is valid for layer thickness *h* below a critical value *h_c_*. This trend disappears when *h* gradually increases to the critical thickness [106]. The size-dependent irradiation hardening in multilayers can be rationalized as follows. Firstly, radiation damage decreases with reducing layer thickness in multilayers [88,89,90,91,92,93,94], as discussed in Section 3. TEM images of He irradiated 50 and 2.5 nm Cu/V multilayers (Figure 17b,c) confirm that 2.5 nm Cu/V multilayers exhibit much better radiation tolerance, as manifested by the formation of low-density of He bubbles and negligible irradiation hardening. Secondly, the size-dependent He irradiation hardening is closely related to the deformation mechanism transition in nanolaminates with reducing layer thickness [128,129,130,131,132]. The deformation mechanisms in multilayers can be classified into three regions with the variation of layer thickness [128,129,130,131,132]. (1) For layer thickness ranging from millimeters to sub-micrometers, dislocation pile-up (Hall–Petch model) in single layer is the dominant deformation mechanism. (2) For layer thickness ranging from hundreds to tens of nanometers, confined layer slip (CLS) is the dominant mode. (3) For layer thickness of several nanometers, dislocation transmitting interface is the main deformation mechanism [128,129,130,131,132]. He irradiation hardening in multilayers with various layer thickness can be well explained based on the three dominant deformation models discussed above. 

**Figure 18:**
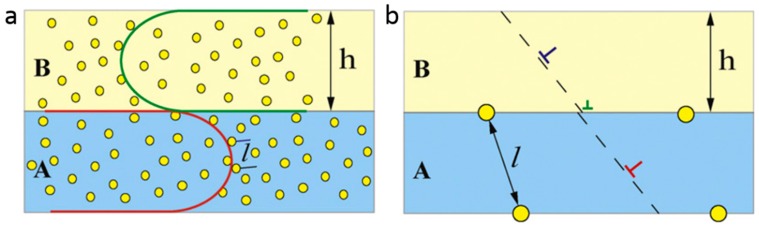
Schematic illustration of dislocation-He bubble interactions in multilayers with various layer thicknesses: (**a**) for large layer thickness, CLS is the dominant deformation mechanism and He bubbles serve as obstacles for dislocations; and (**b**) for small layer thickness, dislocation crossing is the main deformation mechanism and He bubbles on interfaces have a less significant effect on dislocation behavior. Reprinted with permission from [106]; Copyright 2011 Elsevier.

In the cases of dislocation pile-up and CLS, dislocations are expected to cut through bubbles to carry plasticity. Wei et al. [106] proposed that the force of interaction between bubbles and dislocations is confined to a small segment of dislocations. Thus, the total resistant force produced by bubbles can be calculated as the sum of individual interaction force (Figure 18a). The irradiation-induced hardening can be determined by the equilibrium between the line force and sum of resistant force [106]

(5) $$\Delta \tau =\frac{n\tau_{i}bl}{bh^{\prime }}$$

where *n* is the number of bubbles along dislocation, $\tau_{i}$ is the average shear strength of bubble, *b* is the Burgers vector, *l* is bubble spacing, and $h^{\prime }$ is layer thickness parallel to glide plane. Parameter *n* in Equation (5) can be substituted using $n=\left(h^{\prime }-l\right)/l$, and then Equation (5) can be rewritten as

(6) $$\Delta \tau =\tau_{i}\left(1-\frac{l}{\sqrt{2}h}\right)$$

For multilayers with layer thickness *h* ranging from millimeters to sub-micrometers, irradiation hardening derives from bubble–dislocation interactions in single layers. In this region, *h* is far larger than the average bubble spacing *l*. In addition, because *h* is larger than the critical layer thickness *h_c_*, the irradiation hardening is about $\tau_{i}$ [106].

For multilayers with layer thickness *h* ranging from hundreds to tens of nanometers, CLS is the dominant deformation mechanism and bubbles distributing inside layers serve as obstacles for dislocation motion. According to Equation (6), irradiation hardening Δτ decreases with reducing layer thickness *h*, manifested as size-dependent irradiation hardening (Figure 18a).

For multilayers with layer thickness *h* of several nanometers (<5 nm), dislocation transmitting interface serve as the dominant mechanism. For nanolaminates, He bubbles tend to array on interfaces and only some bubbles stay inside layers (Figure 18b). Because Equations (5) and (6) are established based on the situation that bubble–dislocation interactions occur in a single layer (dislocation pile-up and CLS dominate), they are not applicable to calculate He radiation hardening in extremely thin multilayers [64,93]. Li et al. [93] reported that 5 nm and 2.5 nm multilayers after implant of 7 at% He demonstrate similar irradiation hardening as that of after 1 at. % He implantation. This implies that the interface He bubbles/platelet produce a similar hardening effects and only give rise to modest hardening in multilayers containing extremely thin layers [93,96]. The detailed mechanism of interaction between dislocation and He platelet/bubble still needs further study.

### 4.3. Modeling of Helium Radiation Hardening

A widely used model to calculate He irradiation hardening is the Friedel–Kroupa–Hirsch (FKH) model [126,128,133,134]. He irradiation hardening in monolithic metals and metallic multilayers containing thick layers (*h* > 5 nm) can be evaluated as [133,134]

(7) $$\Delta \sigma =\frac{1}{8}M\mu bdN^{2/3}$$

where *M* is the Taylor factor, μ is the shear modulus, *b* is the Burgers vector, *d* is the average bubble diameter, and *N* is the average bubble density. This model [133] was initially presented to describe elastic interactions of dislocations and dislocation loops, but it works well in evaluating hardening induced by weak obstacles for dislocation motion, such as He bubbles [126]. Generally, the FKH model is applicable to obstacles with a barrier strength lower than 0.25 [134]. As He bubbles deliver a barrier strength of about 0.2 [135], this low barrier strength makes it reasonable to calculate He irradiation hardening using the FKH model.

A simplified Orowan model is also proposed to evaluate radiation-induced hardening in metals [93,136,137,138]. The model can be expressed as follow.

(8) Δτ=μb/Kl

where *l* is the average obstacle spacing, *K* is a factor related to obstacle strength. Obviously, strong obstacles usually deliver small *K* [136,137]. Osetsky et al. [136,137] demonstrated that *K* is about 1.8–10 for impenetrable defects depending on obstacle size, and is 2–5 for voids. He bubbles with *l* = 7.9 nm have *K* = 5.5 in Ag/V multilayers. Generally, He bubbles have larger *K* value compared to voids due to relatively low obstacle strength.

He bubbles with low equilibrium pressure usually serve as shearable obstacles for dislocation motion [123]. For shearable bubbles, dislocations are forced to bow out and then cut bubbles to further carry plasticity, as identified in He irradiated Cu [122]. The resolved shear stress required for a dislocation to cut two adjacent He bubbles is calculated using the modified Orowan equation [106,139].

(9) $$\tau =\frac{\mu b}{2\pi l}\ln\left(\frac{l}{r}\right){\left(\cos\varphi_{c}\right)}^{1/2}$$

where μ, b and *l* have the same meaning as in Equations (8) and (9). r is the radius of He bubbles and $\varphi_{c}$ is defined as half of the critical angle of a bow-out dislocation. $\varphi_{c}$ can be regarded as a reference to evaluate dislocations and obstacles interactions. $\varphi_{c}=0^{{}^{\circ}}$ corresponds to strong obstacles and Equation (9) deduces to Orowan formula. Very weak obstacles deliver $\varphi_{c}$ of about 90°. Wei et al. [106] found that $\varphi_{c}=76^{{}^{\circ}}$ for 0.8 nm He bubbles in V/Ag multilayers. Similarly, we calculated that $\varphi_{c}$ is about 60° for 4 nm He bubbles in Al–4Cu alloy [127]. These relatively large $\varphi_{c}$ indicate that shearable He bubbles are weak obstacles for dislocation motion. 

## 5. Effect of Helium Bubbles on Ductility of Metals

### 5.1. High Temperature Helium Embrittlement in Polycrystalline Metals

**Figure 19:**
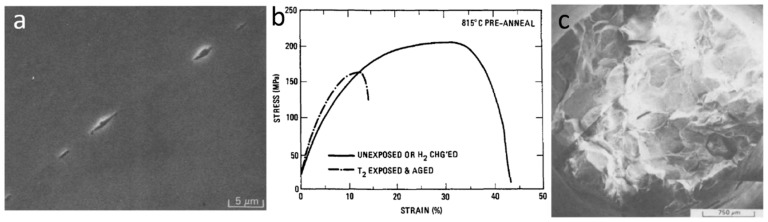
(**a**) Intergranular cavities along GB in Cu after T_2_ exposure; (**b**) tensile behavior of unexposed and T_2_ charged and aged Cu; and (**c**) intergranular fracture surface morphology of Cu after tritium exposure treatment. Reprinted with permission from [140]; Copyright 1986 Elsevier.

As one form of well-known radiation damage, high temperature He embrittlement in metals has attracted tremendous interests in past decades [140,141,142,143,144,145,146,147,148,149,150,151,152,153,154,155,156,157,158,159,160,161,162,163]. A very low overall helium concentration could significantly degenerate the mechanical properties of metals, leading to catastrophic fracture of nuclear reactor components [140,141,142,143,144,145,146,147,148,149,150,151,152,153,154,155,156,157,158,159,160,161,162,163]. Kramer et al. [142] performed tensile tests on 304 SS after He implantation. They reported that an extremely low He concentration, about 1 × 10^−7^ atom fraction of He above 650 °C and 3 × 10^−5^ atom fraction of He above 540 °C resulting in severe ductility loss in 304 SS. A representative work to evaluate He effects on metals was conducted on oxygen free high purity Cu as well [140]. It is found that only about 2 appm He concentration can exert a profound effect on microstructure of Cu [140]. As shown in Figure 19a, a series of intergranular cavities with various size are formed along GB, which originate from coalescence of He bubbles formed along GBs. Specimens containing He introduced by tritium decay also show significant ductility loss (Figure 19b). In addition, the fracture surface (Figure 19c) has an intergranular fracture morphology, presumably due to radiation-induced cavities along GBs (Figure 19a).

**Figure 20:**
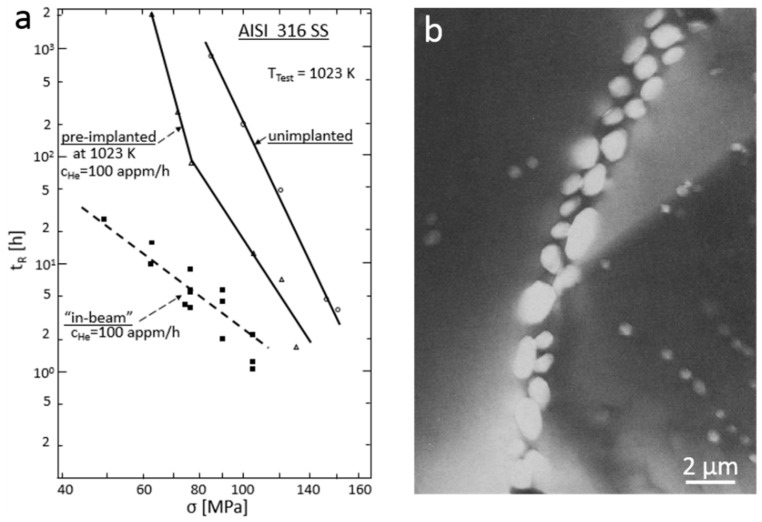
(**a**) The dependence of creep rupture time on creep stress of 316 SS at 1023 K with different irradiation parameters; and (**b**) large He bubbles distributed along GB, which is perpendicular to the applied stress after creep test. Reprinted with permission from [141]; Copyright 1983 Elsevier.

Investigations performed in the past decades have gained valuable insights into high temperature He embrittlement in metals. However, the evolution process of He bubbles under stress that leads to final embrittlement is rather complicated. Stress enhanced bubble growth along GBs is widely accepted as one of dominant failure mechanisms underlying high temperature He embrittlement [141,142,143,144,145,146,147,148,149,150,151], but there still leaves many opening questions. Creep tests on samples after low temperature He implantation or during He implantation (named “in beam” [141]) are regarded as the most instructive way to investigate high temperature He embrittlement, as “in beam” creep tests provide an opportunity to monitor the complete bubble evolution processes under stress. Here, we briefly discuss creep tests [141] performed on 316 SS and DIN 1.4970 SS after room temperature He implantation or during He implantation [141]. Figure 20a plots the dependence of creep rupture time on creep stress in 316 SS at 1023 K. Under the same creep stress, He-irradiated 316 SS delivers much shorter creep rupture time than their counterparts without radiation [141]. Among irradiated 316 SS, the “in beam” samples show the shortest creep rupture time, indicating that high-temperature He irradiation may significantly reduce the service life of metals. In addition, all irradiated samples exhibit brittle, intergranular fracture, while samples without radiation show transgranular cup-cone fracture mixed with ductile tearing [141]. Figure 20b shows typical TEM image of 316 SS “in beam” tests after 25 hours under creep stress of 50 MPa at 1023 K. The corresponding He concentration is 2500 appm. He bubbles with mean diameter of about 100 nm distributed along GB, which is perpendicular to the applied stress. These creep tests further demonstrate that stress-enhanced bubble growth along GBs is responsible for high temperature He embrittlement [141,142,143,144,145,146,147,148,149,150,151].

**Figure 21:**
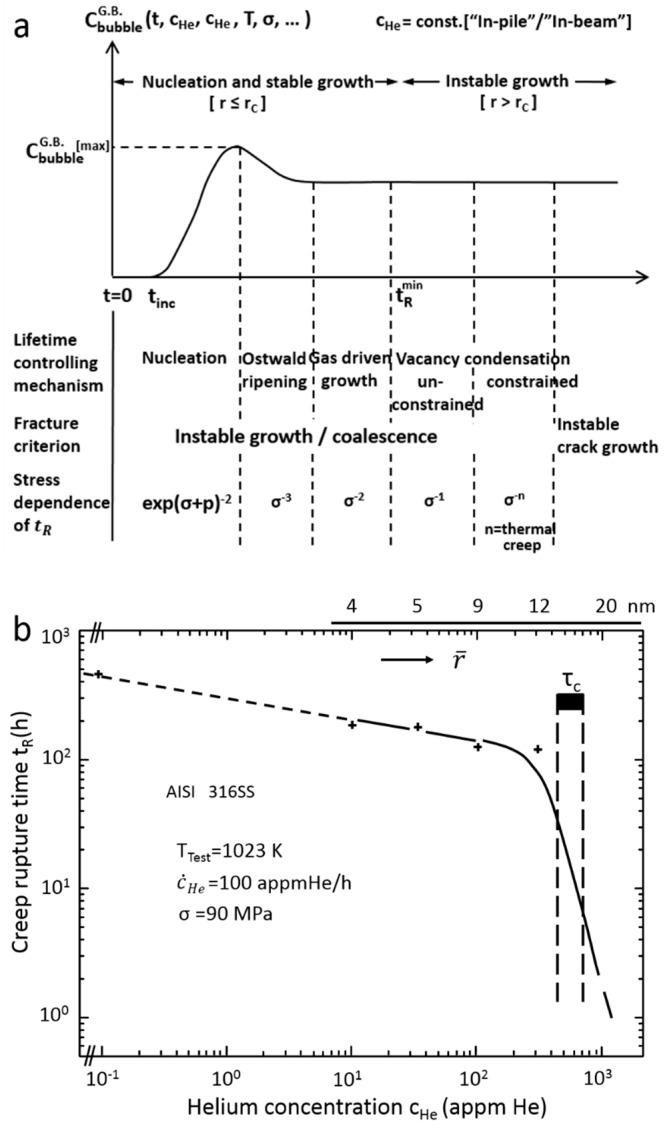
(**a**) The bubble evolution process along GB in creep test [158]; and (**b**) the creep rupture time decreases with increasing He concentration. The creep rupture time decreases sharply when He bubble size reaches the critical size [18]. Reprinted with permission from [158]; Copyright 1983 Informa UK Limited; Reprinted with permission from [18]; Copyright 1985 Elsevier.

Next, we focus on He bubble evolution at high temperature during “in beam” creep tests. As schematically illustrated in Figure 21a, high-temperature He embrittlement consists of a sequence of processes including bubble nucleation, stable gas-driven bubble growth, unstable stress-driven void growth and crack nucleation by cavity coalescence [144,145,146,147,148,149,150,151,158]. “In beam” creep tests are accompanied by a constant He irradiation rate ($\dot{c_{\mathrm{He}}}$). At the very beginning, He forms He–V clusters and develops into He bubbles gradually along GBs by absorbing additional He atoms and vacancies. With further increasing He concentration, the bubble concentration along GBs, $c_{\mathrm{Bubble}}^{\mathrm{GB}}$, will increase, as shown in nucleation part in Figure 21a. $c_{\mathrm{Bubble}}^{\mathrm{GB}}$ reaches a peak value with further increasing of He concentration [151,158]. Thus, bubble nucleation is suppressed and bubble growth is dominant [150]. $c_{\mathrm{Bubble}}^{\mathrm{GB}}$ is supposed to reduce due to bubble growth and coarsening (Figure 21a). Before reaching the critical radius *c_r_*, He bubbles grow in a stable gas-driven growth mode by absorbing He and vacancies at an equilibrium pressure p=2γ/r, where γ is surface energy of matrix and r is radius of He bubbles [56,144,145,146,147,148,149,150,151,158]. However, irradiation or stress-induced effective vacancy supersaturation will enhance the transition from gas-driven bubble growth to unstable stress-driven void growth at an equilibrium pressure p<2γ/r [56,146,147,148,158]. Once the He bubble reaches the critical radius *c_r_*, a transition from bubble-to-void happens, and the samples will fracture in a short time once exposed to creep stress. An important conclusion on creep tests is that creep life time is dominated by gas-driven growth of bubbles along GBs [56,145,146,147]. This indicates that the time from bubble nucleation to bubble–void transition is regarded as a rupture criterion [145,146,147]. After the bubble-to-void transition, voids will grow unstably under creep stress. Finally, voids coalescence leads to crack nucleation and final catastrophic intergranular fracture.

Based on the discussion above, it is obvious that the critical radius *c_r_* plays as an important role in determining the creep life. For GBs containing He bubbles under creep stress, the critical size for bubble-to-void transition can be calculated as [145]

(10) $$c_{r}=4\gamma /3\sigma$$

where γ is surface energy of matrix and σ is the creep stress. When the creep stress is removed, the critical size for bubble-to-void transition is [56,145]

(11) $$c_{r0}=c_{r}/\sqrt{3}=4\gamma /3\sqrt{3}\sigma$$

Below this critical size, He bubbles remain stable and high-temperature He embrittlement will be suppressed. Two practical approaches are proposed to mitigate high-temperature He embrittlement in metals [64]: (1) maximize the critical size for bubble-to-void transition by reducing irradiation-induced vacancies; and (2) maximize bubble nucleation sites to increase the number of stable He bubble. In fact, NC metals [70,71,72,73], multilayers [80,88,89,90,91,92,93,94,95,96,97,98,99,100,101] and oxide dispersion-strengthened (ODS) steels [164,165] are examples designed based on the above two approaches. Abundant interfaces in NC metals, multilayers and ODS steels significantly enhance the recombination of interstitials and vacancies, which reduce the production rate of irradiation-induced vacancies [68,69]. Moreover, these interfaces are preferable nucleation sites for He bubbles [97,98], which suppresses overall bubble growth and keeps He bubble below the critical size for bubble-to-void transition.

### 5.2. Helium Bubbles Enhance Ductility in Small-Volume Single-Crystal Metals

**Figure 22:**
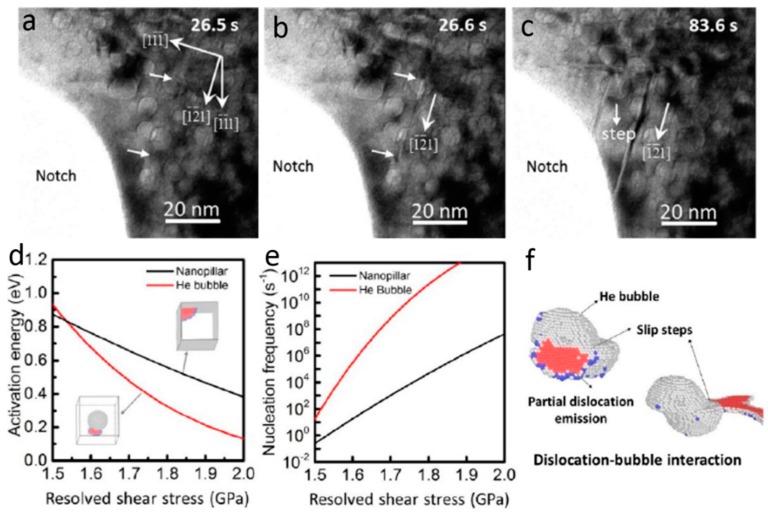
(**a**–**c**) A partial dislocation nucleates from He bubble and cuts through adjacent He bubbles, leaving a stacking fault and slip steps on bubbles. (**d**,**e**) A simulation demonstrates that He bubbles are preferable dislocation nucleation sites. For resolved shear stress higher than 1.5 GPa, the activation energy for dislocation nucleation from He bubbles is lower than that from pillar surface/corner, leading to higher dislocation nucleation frequency from He bubbles. (**f**) Slip steps on He bubbles also serve as dislocation nucleation sites. Reprinted with permission from [122]; Copyright 2016 American Chemical Society.

Contrary to polycrystalline metals, nanoscale He bubbles are demonstrated to enhance ductility in small-volume single-crystal metals [122,127] and metallic glass [166]. The underlying mechanisms are discussed as follows. Firstly, because they serve as shearable obstacles, He bubbles hinder dislocation motion and reduce dislocation mean free path, and hence promote dislocation storage, which gives rise to stable and homogeneous plasticity [122,126,127]. Secondly, He bubbles are preferable internal dislocation nucleation sources [122]. As shown in Figure 22, both experiments and simulations have confirmed this novel deformation mechanism. Figure 22a,c presents snapshots recording dislocation operation in sample containing dense He bubbles in Cu. A partial dislocation nucleates from the adjacent region of He bubble within 0.1 s (Figure 22b). This indicates that the He bubble surface and sample surface are near equal preferable dislocation nucleation sites [122]. Similarly, dislocations are also observed to nucleate and propagate homogeneously across the whole small-volume Al–4Cu sample containing He bubbles [127], which is in sharp contrast to localized dislocation operation in bubble-free sample. Subsequently, He bubbles are cut through by dislocations, as indicated by the steps and stacking faults in Figure 22b,c. Atomic simulations indicate that He bubbles are preferable dislocation nucleation sites compared to pillar surface/corner at the resolved shear stress above 1.5 GPa, which is usually the critical resolved shear stress required for dislocation nucleation in molecular dynamic simulations. Above this critical shear stress, the dislocation nucleation frequency is higher and the activation energy is lower for dislocation nucleation from He bubbles than that of dislocation nucleation from pillar surface/corner (Figure 22d,e). He bubble–dislocation interaction can further produce massive slip steps at He bubble surface, which are new internal dislocation nucleation sources, as shown in Figure 22f. Therefore, He bubbles play a combined role of shearable obstacles and internal dislocation nucleation sources, which promote dislocation storage in small-volume samples, thus enhance stable and homogeneous deformability in SC pillars. In addition, besides bubble coalescence, bubble cleavage is also identified as an alternative micro-damage mechanism in deformation of He-bubbled SC pillars [123]. This provides new insight into the failure mechanism of He-bubbled metals.

The surprising finding that He bubbles enhance ductility in single-crystal metals may provide new applications for ion beam engineering. By deliberately introducing bubbles or other defects using ion beam engineering, mechanically robust single-crystal devices can be manufactured [121]. Moreover, this finding provides a new approach to suppress high temperature He embrittlement in nuclear reactor components. Single-crystal metals may be considered as a potential candidates for nuclear reactor components, but a series of issues, such as recrystallization of deformed SC metals at elevated temperature, the difficulty of fabricating bulk single-crystal metals, etc., remain as challenges. 

## 6. Summary and Outlook

Helium irradiation-induced damages have been studied extensively in the past decades and remain an important issue today. In this review, we briefly summarize previous studies on He bubble evolution and their effects on mechanical properties of metals. He bubble nucleation and growth are promoted with the aid of radiation produced vacancies. Matrix atoms are pushed out and form Frenkel pairs with increasing He atoms in He–V clusters. In addition, He bubbles grow accompanied by dislocation loop punching. Bubble migration and coalescence and Ostwald ripening are two main mechanisms of bubble coarsening under annealing. Interface engineering is widely adopted to improve radiation tolerance of metals. Interfaces serve as sinks for point defects (vacancies, interstitials and He), which promote vacancy–interstitial recombination and assist the stable storage of He. Dense He bubbles act as shearable obstacles under straining, and induce hardening in metals. Multilayers demonstrate size-dependent hardening due to the transition of deformation mechanism with reducing layer thickness. High temperature He embrittlement consists of four stages: bubble nucleation, stable gas-driven bubble growth, unstable stress-driven void growth and crack nucleation by cavity coalescence. Once the bubble size is larger than a critical value, a transition from bubble-to-void takes place and leads to catastrophic intergranular fracture. He bubbles can enhance the strength and ductility in small-volume metals because He bubbles play a combined role of shearable obstacles and dislocation sources. 

As discussed in previous sections, significant progresses have been made in understanding of He bubbles in metals. However, there are still paramount problems left that need to be addressed in the future. In the following, we only propose some of representative issues that need special attention.

First, most nuclear reactor components serve at medium or high temperatures, and the thermal stability of interfaces in NC metals and multilayers should be considered with particular concern. Radiation-assisted grain growth in NC metals and layer pinch-off in multilayers will lead to sharp reduction in the density of interfaces, which dramatically deteriorates radiation tolerance in NC metals and multilayers. There are increasing studies on thermal stability of GBs and heterophase interfaces, but there are still very limited efficient approaches to improve the thermal stability of GBs and heterophase interfaces in metals, especially under radiation condition. Improving the thermal stability of interfaces without sacrificing the mechanical properties will give rise to unprecedented radiation tolerance in NC metals and multilayers.

Second, details of He–interface interactions are still not very clear and need further study. Experimental evidence from previous studies confirm that interfaces are efficient sinks for He, but existing studies do not provide a clear picture on details of He–interface interactions. Atomic simulations may provide an effective way to unveil He–interface interactions, but studies using atomic simulations on this issue are still limited. Moreover, in situ He irradiation inside TEM may serves as a direct and visual method to further investigate this question.

Third, microstructures of GBs and heterophase interfaces are supposed to evolve due to direct irradiation and interactions with point defects induced by collision cascade. As a result, the capacity of interfaces in trapping point defects (He, vacancies and interstitials) and the ability of promoting vacancy–interstitial recombination may alter or reduce with continuous interface–defect interactions. Thus, a method to design self-healing interface is highly desired to further improve the radiation resistance of metals.
